# Assignment by Negative-Ion Electrospray Tandem Mass Spectrometry of the Tetrasaccharide Backbones of Monosialylated Glycans Released from Bovine Brain Gangliosides

**DOI:** 10.1007/s13361-018-1944-8

**Published:** 2018-05-11

**Authors:** Wengang Chai, Yibing Zhang, Laura Mauri, Maria G. Ciampa, Barbara Mulloy, Sandro Sonnino, Ten Feizi

**Affiliations:** 1 Glycosciences Laboratory, Department of Medicine, Imperial College London, Hammersmith Campus, London, W12 0NN UK; 2 Department of Medical Chemistry, Biochemistry and Biotechnology, Center of Excellence on Neurodegenerative Diseases, Graduate School of Biochemical, Nutritional and Metabolic Sciences, University of Milan, 20090 Segrate, Italy

**Keywords:** Gangliosides, Bovine brain, Negative-ion ESI-CID-MA/MS, Glycans, Oligosaccharides, Sequence assignment, Backbone structures

## Abstract

Gangliosides, as plasma membrane-associated sialylated glycolipids, are antigenic structures and they serve as ligands for adhesion proteins of pathogens, for toxins of bacteria, and for endogenous proteins of the host. The detectability by carbohydrate-binding proteins of glycan antigens and ligands on glycolipids can be influenced by the differing lipid moieties. To investigate glycan sequences of gangliosides as recognition structures, we have underway a program of work to develop a “gangliome” microarray consisting of isolated natural gangliosides and neoglycolipids (NGLs) derived from glycans released from them, and each linked to the same lipid molecule for arraying and comparative microarray binding analyses. Here, in the first phase of our studies, we describe a strategy for high-sensitivity assignment of the tetrasaccharide backbones and application to identification of eight of monosialylated glycans released from bovine brain gangliosides. This approach is based on negative-ion electrospray mass spectrometry with collision-induced dissociation (ESI-CID-MS/MS) of the desialylated glycans. Using this strategy, we have the data on backbone regions of four minor components among the monosialo-ganglioside-derived glycans; these are of the *ganglio*-, *lacto*-, and *neolacto*-series.

**Electronic supplementary material:**

The online version of this article (10.1007/s13361-018-1944-8) contains supplementary material, which is available to authorized users.

**Figure:**
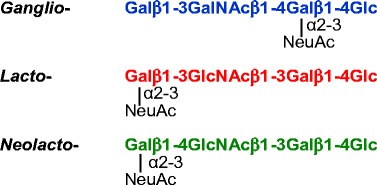
Graphical abstract

## Introduction

Gangliosides are plasma membrane-associated and sialylated glycolipids. The glycan moieties constitute cell surface antigens; also among them are ligands for endogenous carbohydrate-recognition proteins, host cell attachment sites for viruses, bacteria, and parasitic agents, and toxins of bacteria. Certain lipid moieties can influence the orientation and presentation of facets of the glycans such that they may not always be accessible for binding by particular carbohydrate-recognizing proteins [1, 2]. There are occasions when the assignment of the glycan antigens and ligands on glycolipids require deconvolution of the glycan and lipid moieties [3].

HPLC separation and purification, and sequence determination by mass spectrometry (MS) and NMR of glycolipids can be difficult due to the presence of multiple lipids. To investigate gangliosides as recognition structures, we have underway a program to develop a “gangliome” microarray using both natural gangliosides and neoglycolipids (NGLs) [4, 5] derived from glycans that are released from them. In the NGLs, each glycan is linked to the same lipid molecule and arrayed in a liposomal formulation for comparative microarray binding analyses. This is a different strategy from that of Cummings and colleagues [6] for generating covalently immobilized glycan microarrays.

The glycans of gangliosides can be released from their lipids enzymatically or chemically. We favor ozonolysis followed by alkaline hydrolysis [7] as there is a high yield of glycans irrespective of glycan sequence. The released glycans can be fractionated by HPLC and sequenced by MS and NMR. In our NGL approach, the glycans, once purified and characterized, are conjugated to an amino-phospholipid and converted for arraying and binding studies in comparison with the natural glycolipids, using advanced microarray methodology [4]. Each NGL obtained in this way not only contains a single carbohydrate sequence but also a single lipid chain in order to facilitate assignment of carbohydrate binding specificities. This is a strategy already used successfully in assignment of the preferred *N*-glycolyl over *N*-acetyl GM1 for recognition by Simian virus 40 [3].

Our preliminary HPLC analyses of ganglioside-derived glycans from the mono-, di-, tri-, and tetra-sialylated fractions (GM, GD, GT, and GQ, respectively), obtained by group separation using anion exchange, showed that each fraction contained a few dominant components and many minor components. Among the 188 ganglioside-derived glycan structures reviewed in 2004 by Yu and colleagues, 29 sequences of monosialylated gangliosides were reported as the *ganglio*-series from various tissues of different animals [8, 9]. In bovine brain, there occur gangliosides with other glycan backbones, e.g., the *lacto*- and *neolacto*-series [9] in addition to the *ganglio*-series. Thus, the enormous diversity of carbohydrate structures in gangliosides poses difficulties in separation and characterization. As the quantity obtainable can be too low for conventional NMR analysis, a high-sensitivity MS-based method is highly desirable. In this presentation, we explore the negative-ion electrospray mass spectrometry with collision-induced dissociation (ESI-CID-MS/MS) method, previously developed for both neutral [10, 11] and sialylated oligosaccharides [12], for assignment of the glycan backbone sequences of monosialylated gangliosides.

Here, we describe a new strategy for backbone assignment of monosialylated oligosaccharides released from bovine brain gangliosides. This takes advantage of the wealth of sequence information obtainable from neutral oligosaccharides by negative-ion ESI-CID-MS/MS after chemical desialylation. Using this strategy, we have identified the major and some of the very minor components of bovine brain gangliosides. The results indicate that the diversity of carbohydrate structures of gangliosides in bovine brain is more complex than anticipated.

## Experimental

### Materials

Sialylated pentasaccharide standards LSTa, LSTb, and LSTc were purchased from Dextra Laboratories (Reading, UK), and GM1a and LSTd from Elicityl (Grenoble, France). Trifluoroacetic acid (TFA) of ReagentPlus grade was from Sigma-Aldrich (Gillingham, UK). All solvents used were of HPLC grade.

### Monosialylated Oligosaccharides from Bovine Brain Glycolipids

Oligosaccharides were released from total bovine brain glycolipids by ozonolysis followed by alkaline hydrolysis as described [7]. In brief, bovine brain glycolipids were dissolved at 5 mg/ml in methanol and subjected to ozonolysis as described. Triethanolamine was added to adjust the pH to 10 and the reaction mixture was kept at ambient temperature for 24 h. The released glycans were fractionated by DEAE-Sepharose chromatography to obtain the monosialylated glycan fraction.

### HPLC Fractionation

Individual monosialylated oligosaccharides were isolated by hydrophilic interaction liquid chromatography (HILIC) on an amide column (3.5 μm, 4.6 × 250 mm, XBridge, from Waters, Manchester, UK). Elution was performed with a linear gradient of CH_3_CN/H_2_O (solvent A, 70:30, solvent B, 20: 80, by vol) containing 15 mM KH_2_PO_4_, from 3 to 8% B over 50 min at a flow rate of 1 ml/min, monitored by UV at 196 nm [13]. Fractions were desalted by gel filtration on a Superdex Peptide column (GE Healthcare Life Sciences, Little Chalfont, UK) with elution by NH_4_OAc (0.2 M) at a flow rate of 0.2 ml/min with online detection by refractive index.

Pooled HILIC fractions were further purified by HPLC using a porous graphitized carbon (PGC) column (Hypercarb, 5 μm, 4.6 × 30 mm, from Hypersil, Runcorn, UK). A gradient of acetonitrile was used for elution (solvent A, H_2_O; solvent B, CH_3_CN/H_2_O H_2_O/acetonitrile 20:80; both containing 0.05% TFA; 5–17% B in 35 min) at a flow rate of 1 ml/min, with detection by UV at 206 nm.

Quantitation based on hexose content was by dot-orcinol assay as described [14].

### Desialylation by Mild Acid Hydrolysis

Removal of sialic acid from the sialylated glycans was carried out by a chemical method using TFA [15]. Briefly, a 1 nmol glycan in 10 μl solution was added with 1 μl of 10% TFA. The solution was kept at 80 °C for 45 min before it was dried under a N_2_ stream. To ensure complete removal of TFA, a further 20 μl of water was added and the solution was dried again under a N_2_ stream. The desialylated glycan was dissolved in 50 μl and 1 μl was used for ESI-MS and CID-MS/MS.

### Electrospray Ionization Mass Spectrometry

Negative-ion ESI-MS and collision-induced dissociation (CID) MS/MS were carried out on a Q-TOF or a Synapt G2 mass spectrometer (Waters, Manchester, UK). Nitrogen was used as desolvation and nebulizer gas at a flow rate of 250 and 15 l/h, respectively. Source temperature was 80 °C and the desolvation temperature 150 °C. The capillary voltage was maintained at 3 kV. A cone voltage of 50–80 V was used for CID-MS/MS. A scan rate of 1 s/scan was employed for CID-MS/MS experiments and the acquired spectra were summed for presentation.

Product-ion spectra were obtained from CID with argon as the collision gas at a pressure of 1.7 bar. The collision energy was adjusted between 16 and 38 V for optimal fragmentation for the tetra- to heptasaccharides. For analysis, oligosaccharides were dissolved in H_2_O at a concentration of 10–20 pmol/μl, of which 1 μl was loop-injected. Solvent (CH_3_CN/2 mM NH_4_HCO_3_ 1:1) was delivered by a syringe pump at a flow rate of 10 μl/min.

## Results and Discussion

### Isolation and Characterization of Sialylated Glycans from Bovine Brain Gangliosides

**Figure 1:**
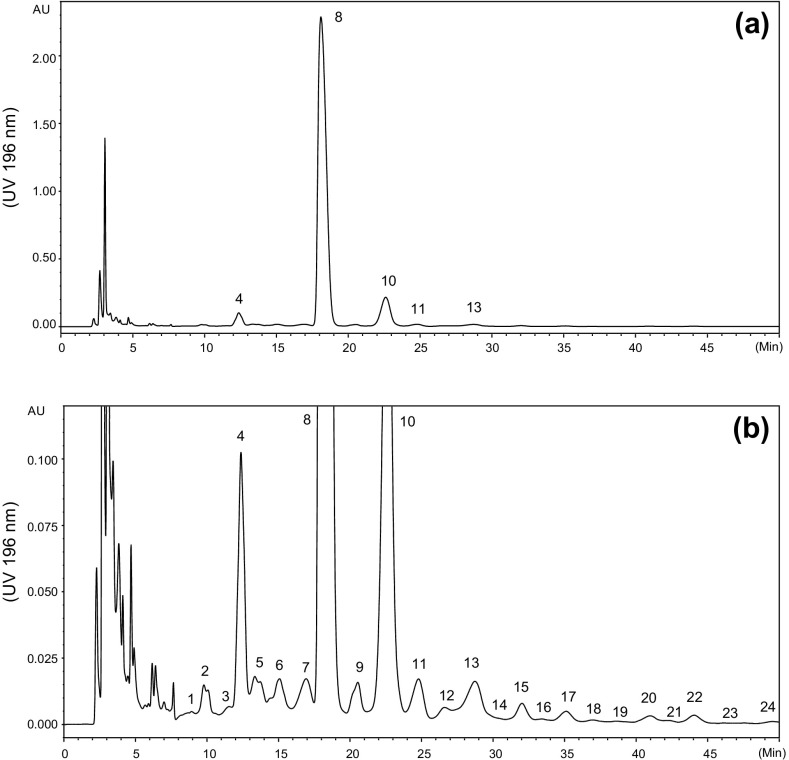
Fractionation of monosialylated glycans obtained from bovine brain gangliosides after cleavage of the ceramide chains by HILIC (amide) and anion-exchange. (**a**) HILIC profile and (**b**) expanded view (intensity × 20)

Sialylated glycans were released from extracted total bovine brain glycolipids by ozonolysis and alkaline hydrolysis. Monosialylated fraction was isolated from these glycans by anion exchange. Further separation of the monosialylated oligosaccharides was carried out by HILIC on an amide column. As shown in Figure 1a, fraction 8 was the dominant component (83.6%); fractions 4 (2.5%) and 10 (9.4%) were also apparent. In an expanded view, 24 fractions were revealed (Figure 1b); the majority is very minor, and these represent less than 4.5% of the total.

**Figure 2:**
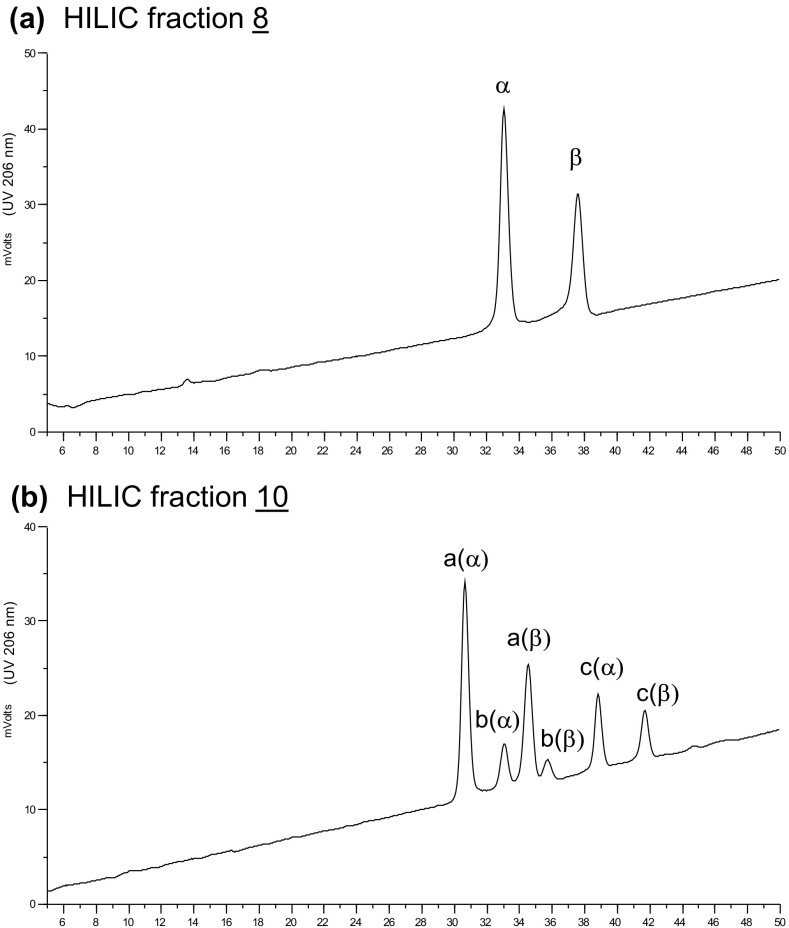
Further fractionation of HILIC fractions 8 (**a**) and 10 (**b**) by PGC-HPLC

Selected HILIC fractions were subjected to further HPLC analysis on a PGC column. The chromatogram obtained for fraction 8 indicated a pure component with well-separated *α*- and *β*-forms as predicted [16]; the *α*-anomer at 33.0 min and *β*-anomer at 37.7 min (Figure 2a). However, fraction 10 showed three components: 10a, 10b, and 10c, each with *α*- and *β*-anomers resolved (Figure 2b).

The multiplicity of the components among the monosialylated glycans of the brain gangliosides and the small amounts of materials available pose a considerable challenge to structural elucidation. Conventional methods such as NMR are not possible. Initial negative-ion ESI-CID-MS/MS was unsuccessful as the product-ion spectra were dominated by desialylation and the very weak fragment ions obtained were too complex to be used for sequence assignment (Suppl Figures 1–3). Therefore, a new MS-based approach was required.

### Unique Fragmentation Patterns for Different Tetrasaccharide Backbone Sequences

As neutral reducing glycans can produce reliable and very informative fragmentation [10, 17], microscale chemical desialylation was carried out before negative-ion ESI-CID-MS/MS. The sialylation information is lost but important backbone sequence information is available. Five monosialylated glycans (Table 1) were selected as reference compounds to establish the fragmentation.

**Table 1:**
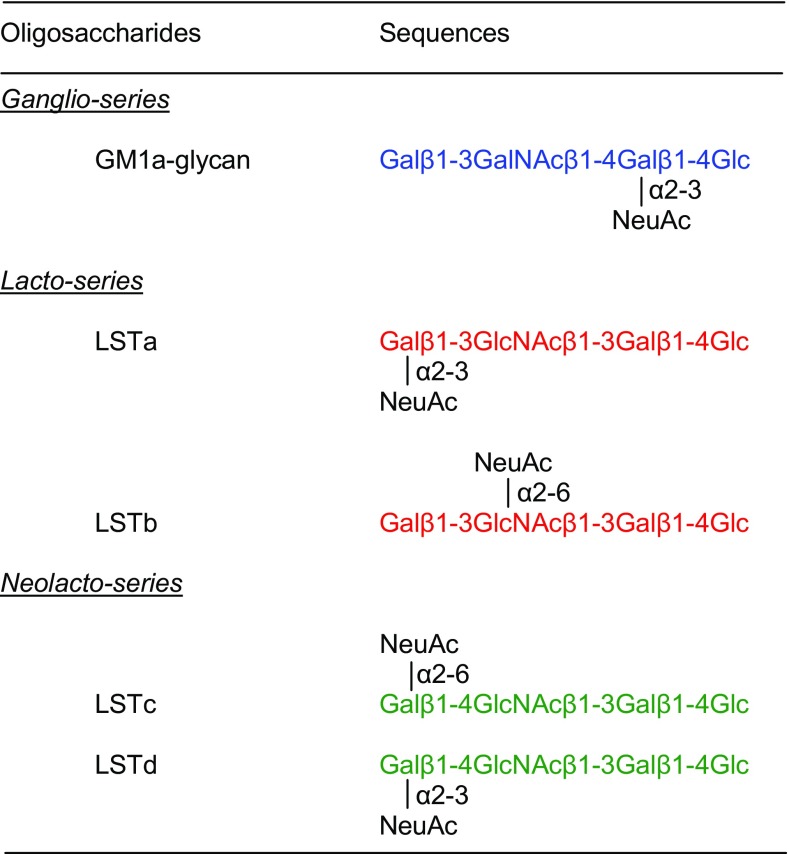
Monosialylated Oligosaccharides Used to Establish Negative-Ion ESI-CID-MS/MS Fragmentation

The colors highlight the different backbone structures: *ganglio-series* in blue, *lacto-series* in red, and *neolacto-series* in green

GM1a contains the *ganglio*-tetrasaccharide sequence Gal1-3GalNAc1-4Gal1-4Glc, and LS tetrasaccharides LSTa and LSTb contain the *lacto*-sequence, Gal1-3GlcNAc1-3Gal1-4Glc, whereas LSTc and LSTd contain the *neolacto*-sequence Gal1-4GlcNAc1-3Gal1-4Glc. Apart from the different internal HexNAc residues (GalNAc in the *ganglio*-series and GlcNAc in the four LS tetrasaccharides), the main difference among the three tetrasaccharide backbones is the difference in the glycosidic linkages, “*3-4-4*” for the *ganglio*-, “*3-3-4*” for the *lacto*-, and “*4-3-4*” for the *neolacto*-series.

**Figure 3:**
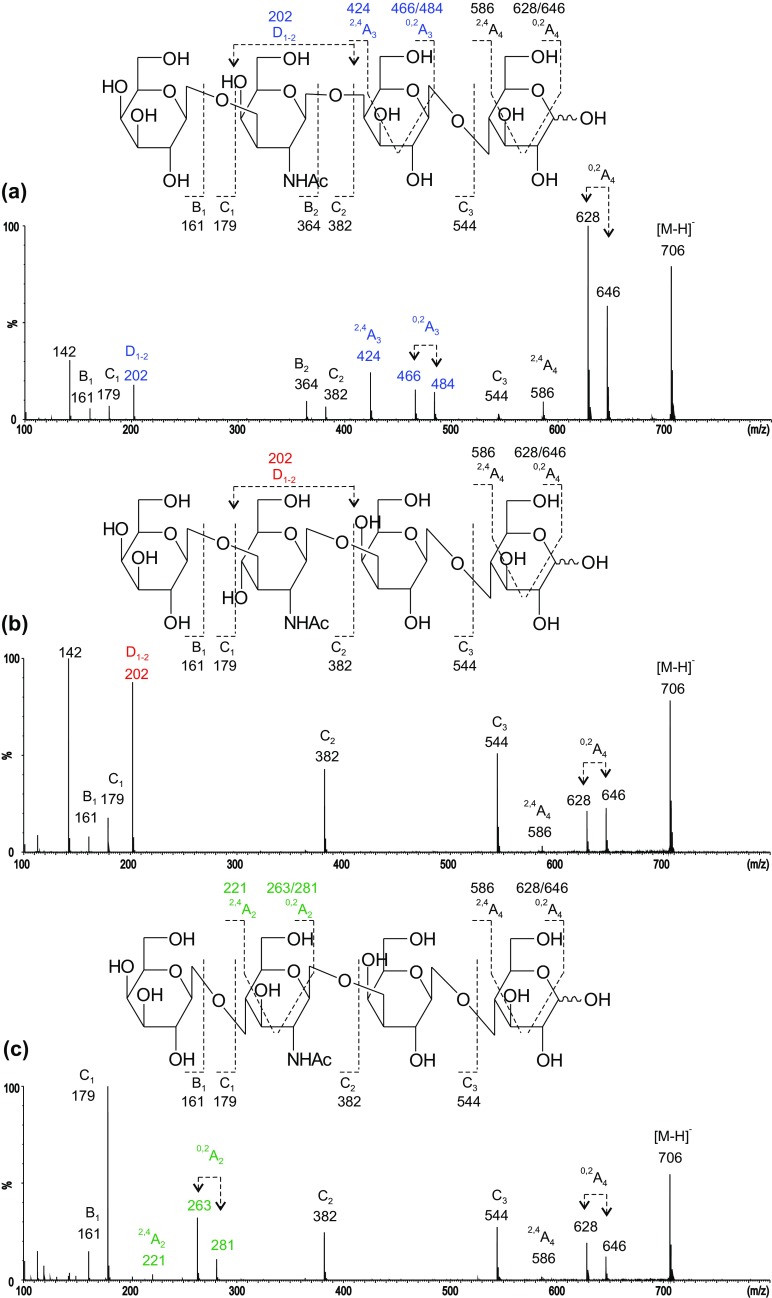
Negative-ion ESI product-ion spectra of tetrasaccharide backbones of the *ganglio*- (**a**), *lacto*- (**b**), and *neolacto*-series (**c**)

Negative-ion ESI-CID product-ion spectra (Figure 3) can be used to identify the backbone sequences via detailed linkage assignment. All three backbone structures gave glycosidic C-ions (C_1_ at *m*/*z* 179, C_2_ at *m*/*z* 382, and C_3_ at *m*/*z* 544) that indicate a linear tetrasaccharide sequence. As all three have a 4-linked Glc at the reducing end, their reducing terminal fragmentations, ^2,4^A_4_ at *m*/*z* 586 and ^0,2^A_4_, together with its dehydrated satellite, at *m*/*z* 646/628, are identical to the three backbone structures. These three A-type fragment ions with neutral loss of − 60/78/120 from the molecular ion [M–H]^−^ at *m*/*z* 706 are characteristic of a 4-linked Hex residue [11].

In the spectrum of desialylated GM1a glycan (Figure 3a), a further set of fragments with neutral loss of − 60/78/120 from C_3_ (*m*/*z* 544) at *m*/*z* 424/466/484 (^2,4^A_3_ and ^0,2^A_3_) is observed for the second residue from the reducing end of this “*3-3-4*” linked tetrasaccharide. In the spectrum of LSTa, this additional set of − 60/78/120 was absent as the internal Gal is 3-linked and this linkage is known not to produce A-type fragments [11] (Figure 3b). As the internal GlcNAc is 3-linked, a double glycosidic cleavage was observed to give a D_1–2_ ion at *m*/*z* 202 as previously reported [10]. The tetrasaccharide backbone structure of LSTb after desialylation is identical to that of LSTa, and the spectrum is the same as that shown in Figure 2b. LSTc and LSTd have the linkage pattern of “*4-3-4*” and therefore the internal 4-linked GlcNAc gave the characteristic ^2,4^A- and ^0,2^A-triple ion set. However, for HexNAc rather than Hex, these are − 101/119/161 rather than − 60/78/120 due to the -NHAc at the C-2-position instead of –OH (a 41 Da difference) and appeared at *m*/*z* 221/263/281 (Figure 3c).

Clearly, the three tetrasaccharide backbone structures, the *ganglio*-, *lacto*-, and *neolacto*-series, gave uniquely different product-ion spectra which can be used for unambiguous assignment of the three different tetrasaccharide backbone regions. With this background, for the assignments of the backbone regions of the glycans released from bovine brain gangliosides, we are making the assumption that the inner monosaccharide HexNAc in the “*3-4-4*” backbones is GalNAc and that in the “*3-3-4*” and the “*4-3-4*” it is GlcNAc.

### Assignment of Tetrasaccharide Backbones of Monosialylated Ganglio-Series Glycans Obtained from Bovine Brain Gangliosides

**Figure 4:**
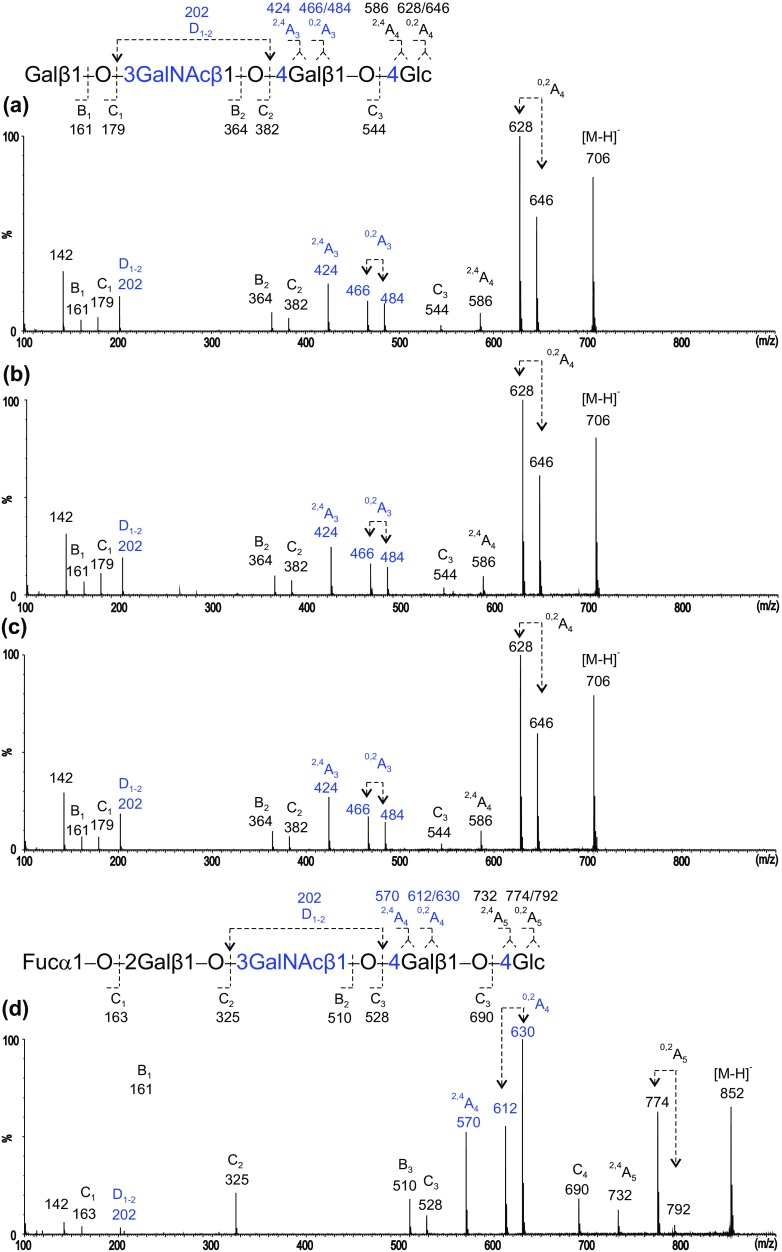
Negative-ion ESI product-ion spectra of desialylated glycans with the *ganglio*-backbones obtained from bovine brain gangliosides. (**a**) GM1a, (**b**) GM1a(Gc), (**c**) GM1b, and (**d**) Fuc-GM1a

Molecular ions of three main sialyl glycans, fractions 8, 10c, and 10a, released from the monosialylated bovine brain gangliosides, are given in Table 2. Those of fractions 8 and 10c are consistent with NeuAc content, and that of 10a with NeuGc. The product-ion spectra of the three glycans after desialylation are shown in Figure 4a–c, respectively, and these are identical and correspond to the presence of “*3-4-4*” linkages, the same as those of the GM1a pentasaccharide standard.

**Table 2:**
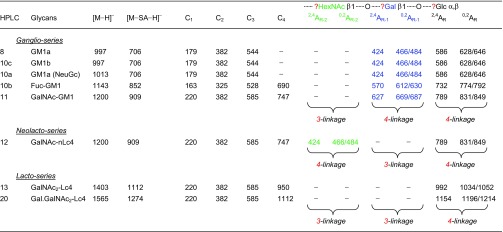
Negative-Ion ESI-MS of Monosialylated Glycans Obtained from Bovine Brain Glycolipids and CID-MS/MS After Desialylation to Assign the Backbone Sequences

[M–H]^−^: deprotonated ion identified by ESI-MS of monosialyl glycolipid-derived glycans; [M–SA–H]^−^: deprotonated ion identified by ESI-MS of monosialylated after chemical desialylation, and used as the precursor ion for CID-MS/MS

Fraction 10b has a [M–H]^−^ ion at *m*/*z* 1143 (Table 2), indicating the presence of an additional monosaccharide (deoxyhexose) taken as Fuc. After desialylation, the product-ion spectrum showed a linear pentasaccharide sequence with the (deoxyhexose) Fuc at the non-reducing end as indicated by the C-type ions (Figure 4d). In the pentasaccharide sequence, the 4-linked Gal next to the reducing Glc is apparent by the set of − 60/78/120 of the ^2,4^A_4_ and ^0,2^A_4_ ions at *m*/*z* 570/612/630. Therefore, fraction 10b can be assigned as having a *ganglio*-backbone sequence with the “*3-4-4*” linkage pattern (Tables 2 and 3) corresponding to the carbohydrate sequence identified as fucosyl GM1 [18].

**Table 3:**
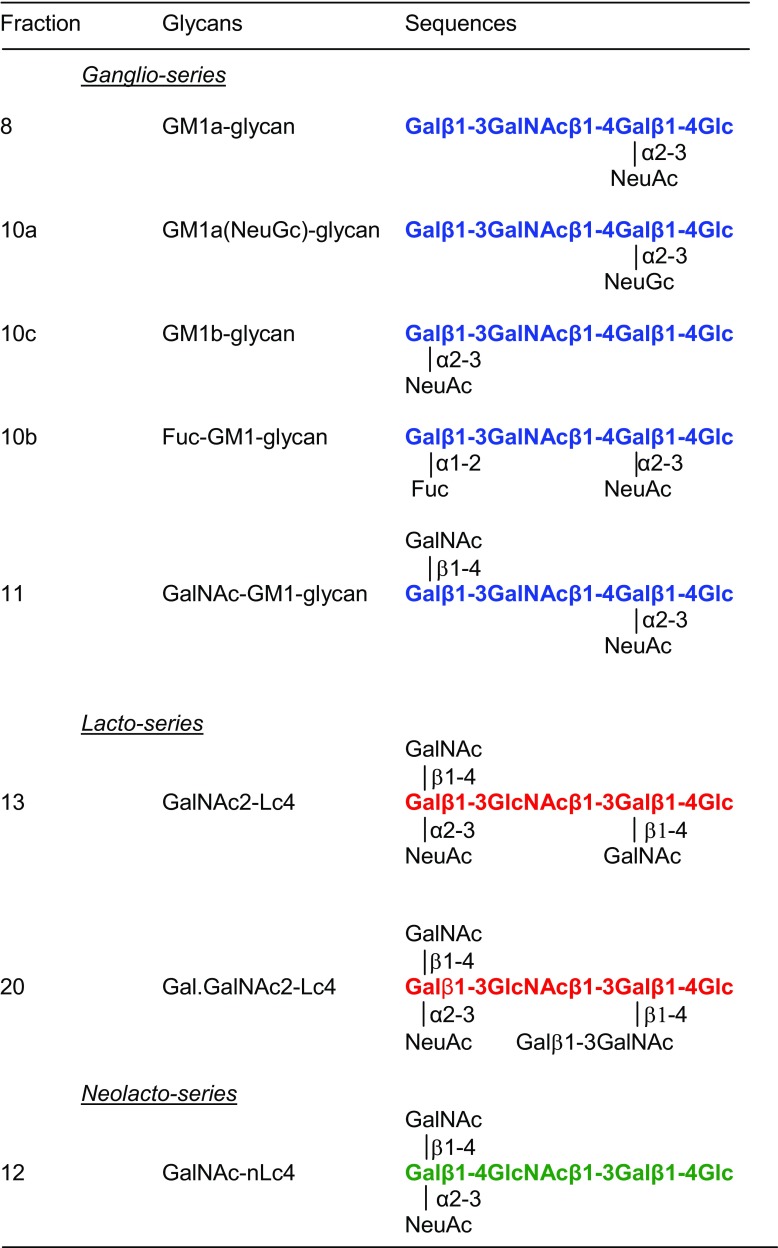
Backbone Sequences Identified by ESI-CID-MS/MS and the Proposed Monosialylated Glycans from Bovine Brain Gangliosides

Backbone sequences of eight glycans released from monosialylated bovine brain glycolipids and assigned by ESI-CID-MS/MS after desialylation, depicted in bold font. The colors depict the *ganglio*-series in blue, *neolacto*-series in green, and *lacto*-series in red. The tentative assignments of the anomeric configuration, positions, and linkages of substituent residues, NeuAc, NeuGc, Fuc, and GalNAc, are based on previous knowledge (see references cited within “Results and Discussion”)

### Assignment Isomeric of Pentasaccharides with *Ganglio*- and *Neolacto*-Backbones from Bovine Brain Gangliosides

**Figure 5:**
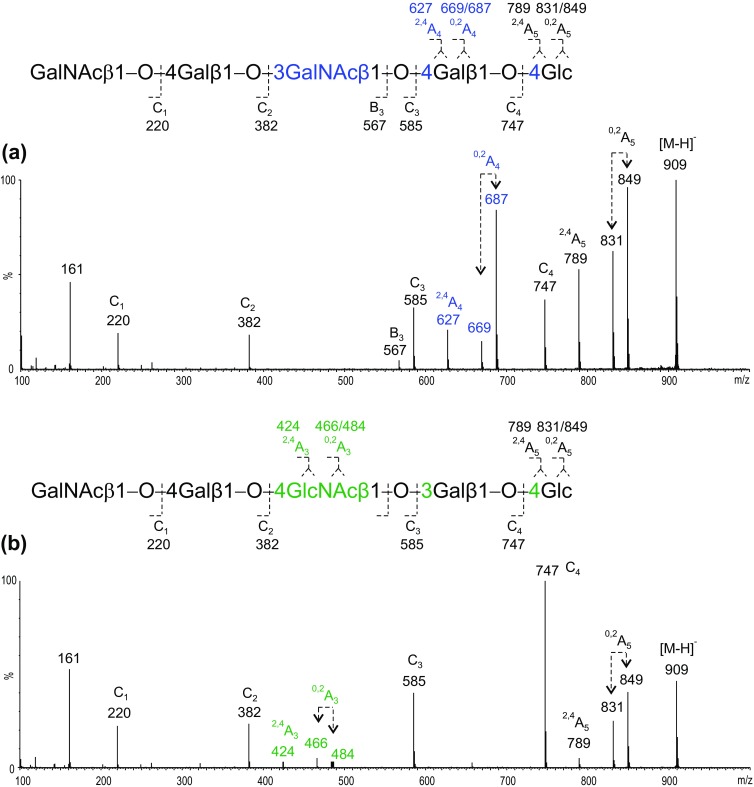
Negative-ion ESI product-ion spectra of isomeric desialylated pentasaccharides with the *ganglio*- (**a**) and *neolacto*-backbones (**b**) obtained from bovine brain gangliosides

The product-ion spectrum of the desialylated fraction 11 (Figure 5a) resembled that of Fuc-GM1 (fraction 10b, Figure 4d) with respect to the *ganglio*-backbone with “*3-4-4*” linkages, characterized by the 4-linked Gal and 4-linked Glc, as shown by the two sets of − 60/78/120 of the ^2,4^A_4_/^0,2^A_4_ and ^2,4^A_5_/^0,2^A_5_ ions at *m*/*z* 627/669/687 and 789/831/849, respectively, in the spectrum. The extended GalNAc was tentatively assigned as β1-4 linked to the Gal as has been shown in GalNAc-GM1 isolated as a very minor brain ganglioside in human brain [19].

The desialylated fraction 12 gave a different spectrum (Figure 5b), which is reminiscent of that of the *neolacto*-series shown in Figure 3c. The main difference between the spectra of fractions 11 and 12 is the internal 4-linked HexNAc. The fragment ion set at *m*/*z* 424/466/484 arising from neutral loss of − 101/119/161 from the C_3_ ion (*m*/*z* 585) identified a 4-linked HexNAc as in the case of LSTc and LSTd with a “*4-3-4*” linkage (Table 2). The extended HexNAc was considered as GalNAc β1-4 linked to the Gal as the carbohydrate sequence with *neolacto*-backbone (Table 3) of glycoprotein gangliosides that express the Sda antigen [20].

### Assignment of Minor Components with Longer Chains of the *Lacto*-Backbone Type

**Figure 6:**
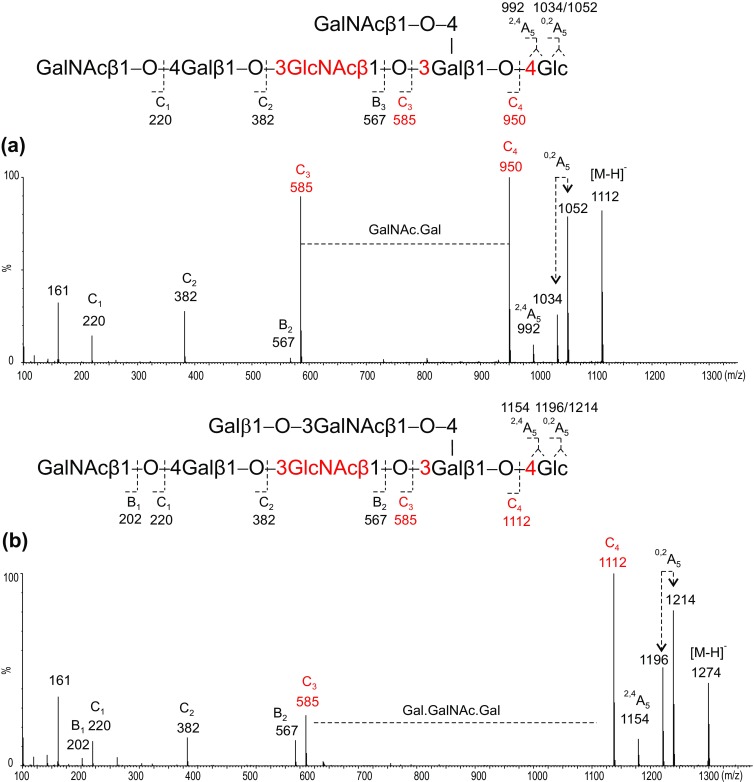
Negative-ion ESI product-ion spectra of desialylated longer chain glycans with *lacto*-backbones obtained from bovine brain gangliosides. (**a**) GalNAc2-GM1 and (**b**) Gal.GalNAc2-GM1

Fractions 13 and 20 are larger glycans with extra HexNAc2 (fraction 13) and Hex1.HexNAc2 residues (fraction 20) on the tetrasaccharide backbones as evidenced by [M–H]^−^ ions at *m*/*z* 1402 and 1565, respectively. In the product-ion spectrum of fraction 13 (Figure 6a), the C-ions at *m*/*z* 220 (C_1_), 382 (C_2_), and 585 (C_3_) clearly identified a linear HexNAc-Hex-HexNac sequence. The gap of 365 Da between C_3_ (*m*/*z* 585) and C_4_ (*m*/*z* 950) indicated a HexNAc branch at the non-reducing end. The lack of other ^2,4^A/^0,2^A-type ions in the spectrum, apart from the reducing terminal 4-linked Glc, suggested a “*3-3-4*” linkage pattern, and therefore, a tetrasaccharide *lacto*-backbone can be proposed for fraction 13 (Tables 2 and 3).

The sequence of fraction 20 that can be similarly proposed as the fragmentation (Figure 6b) is very similar to that of fraction 13. The only difference is the longer gap, 527 Da, between C_3_ (*m*/*z* 585) and C_4_ (*m*/*z* 1112) which indicated a Hex.HexNAc branch at the internal Gal. Again, the lack of other ^2,4^A/^0,2^A-type ions indicated internal “*3-3-4*” linkage pattern; thus, a tetrasaccharide *lacto*-backbone (Tables 2 and 3) consistent with the glycan structures in gangliosides designated X1 and X2 isolated previously from bovine brain [21].

## Conclusions

The [M–H]^−^ ions before and after desialylation provided information on the sialic acid type. By negative-ion ESI-CID-MS/MS [10, 17] analyses after desialylation, a wealth of sequence and linkage information is obtained at high sensitivity (1 pmol) on the backbone sequences of seven of the minor components among the monosialylated glycans released from bovine gangliosides. However, after chemical desialylation, the information on the sialylation site is lost. Conversion of the carboxyl group of sialic acid into neutral functionalities by esterification or amidation will be a way forward to overcome the disadvantage of the present approach.

Fraction 10a was the most abundant in fraction 10. It is the NeuGc analogue of GM1a which is worthy of comment. This sialic acid form is reported as lacking in nervous tissue of animals; its presence in the brain ganglioside extract may represent an origin from non-neural cell types [22]. This can be resolved in due course with immuno-histochemical studies. Fuc-GM1 identified here (fraction 10b) was described as accounting for 1% of gangliosides extracted from bovine brain [23] and it occurs at higher levels in the nervous tissue of mini-pig [18].

Fraction 12 is a *neolacto*-type of glycan, bearing the blood group Sda carbohydrate, GalNAcβ1-4(NeuAcα2-3)Galβ1-4GlcNAc, which is abundantly expressed on glycolipids and glycoproteins in the normal gastrointestinal tract mucosa in humans, but not to our knowledge among brain gangliosides [22].

The proposed structures of fractions 13 and 20 (Table 3) are those of *lacto*-type glycans and correspond to gangliosides designated X1 and X2, isolated and characterized as unique lacto gangliosides, isolated previously from bovine brain [21].

The results give insights into the diversity of glycan structures present in gangliosides of animal brains. They raise questions as to the cellular origins of these.

## Electronic Supplementary Material

ESM 1 (PDF 2575 kb)
