# Supplementary material for: Assignment by Negative-Ion Electrospray Tandem Mass Spectrometry of the Tetrasaccharide Backbones of Monosialylated Glycans Released from Bovine Brain Gangliosides
Source: J Am Soc Mass Spectrom. 2018 May 11;29(6):1308–18. doi: 10.1007/s13361-018-1944-8 (PMC6003998; doi:10.1007/s13361-018-1944-8)
Supplement: Supplementary file 1 — (PDF 2575 kb) [file 13361_2018_1944_MOESM1_ESM.pdf]

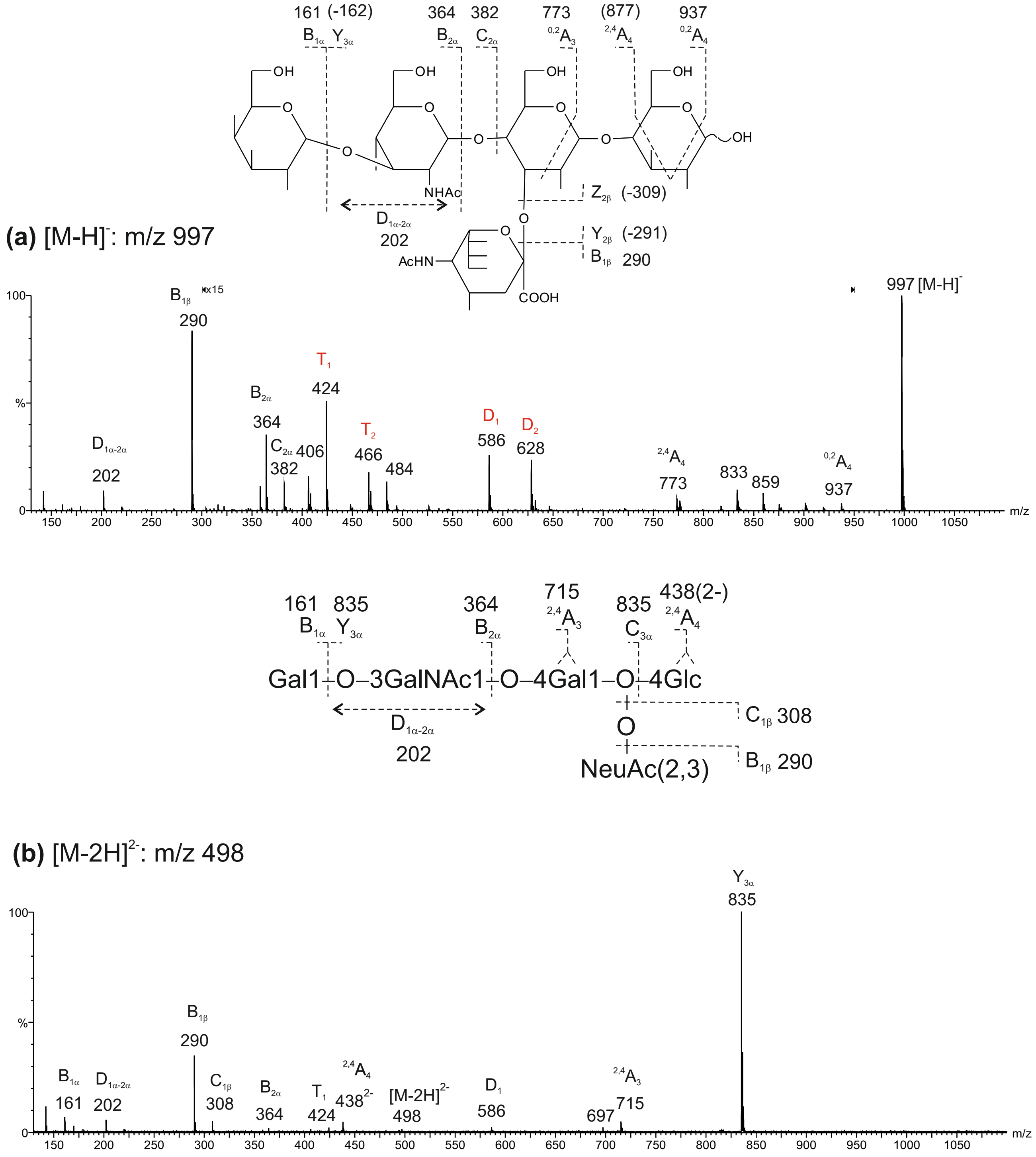

Supplementary Figure 2. ESI-CID-MS/MS of intact GM1a-glycan using [M-H]⁻ (a) and M-H]$^{2-}$ (b) as the precursor ions.

Please note the magnification factor x15 used in spectrum (a) for the region of m/z 300-950.

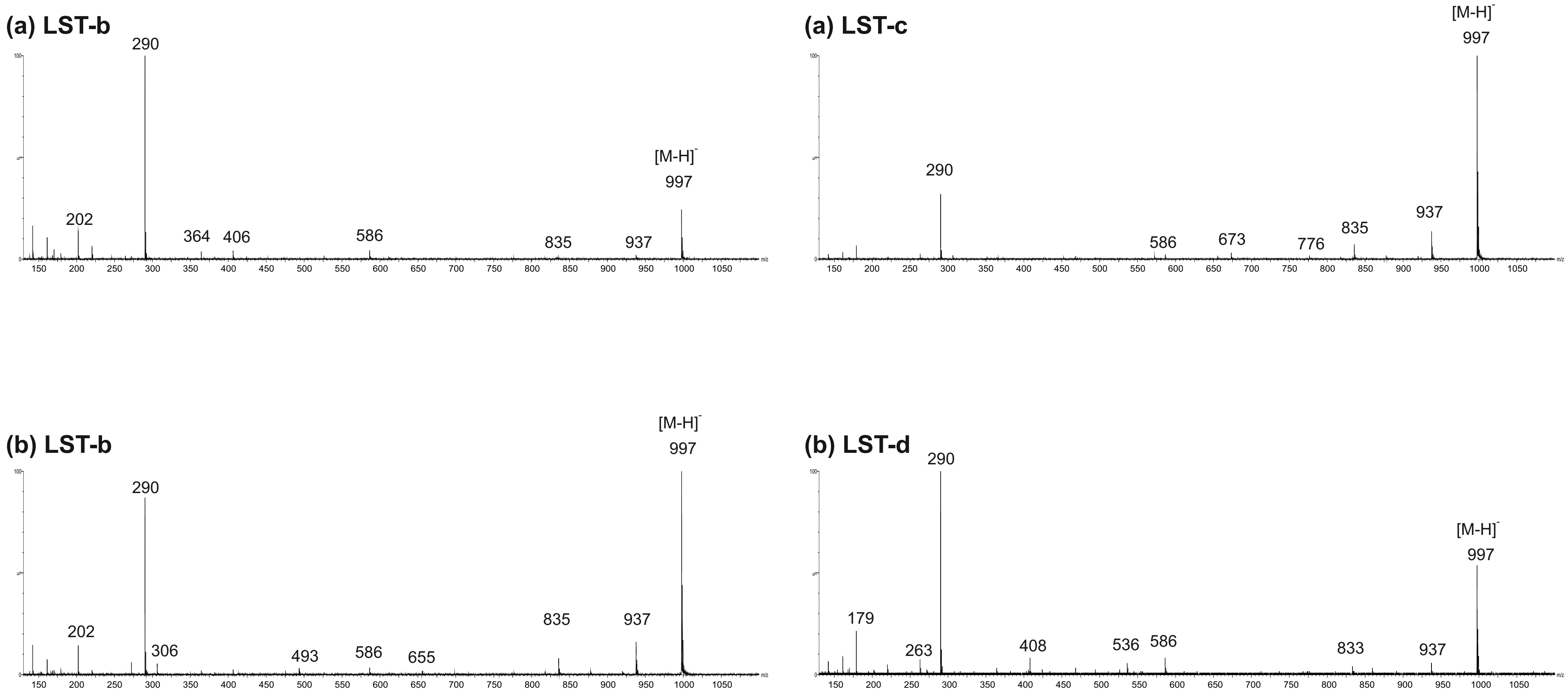

Supplementary Figure 2. ESI-CID-MS/MS of intact LSTa (a), LSTb (b), LSTc (c) and LSTd (d) using $[M-H]^-$ as the precursor ions.

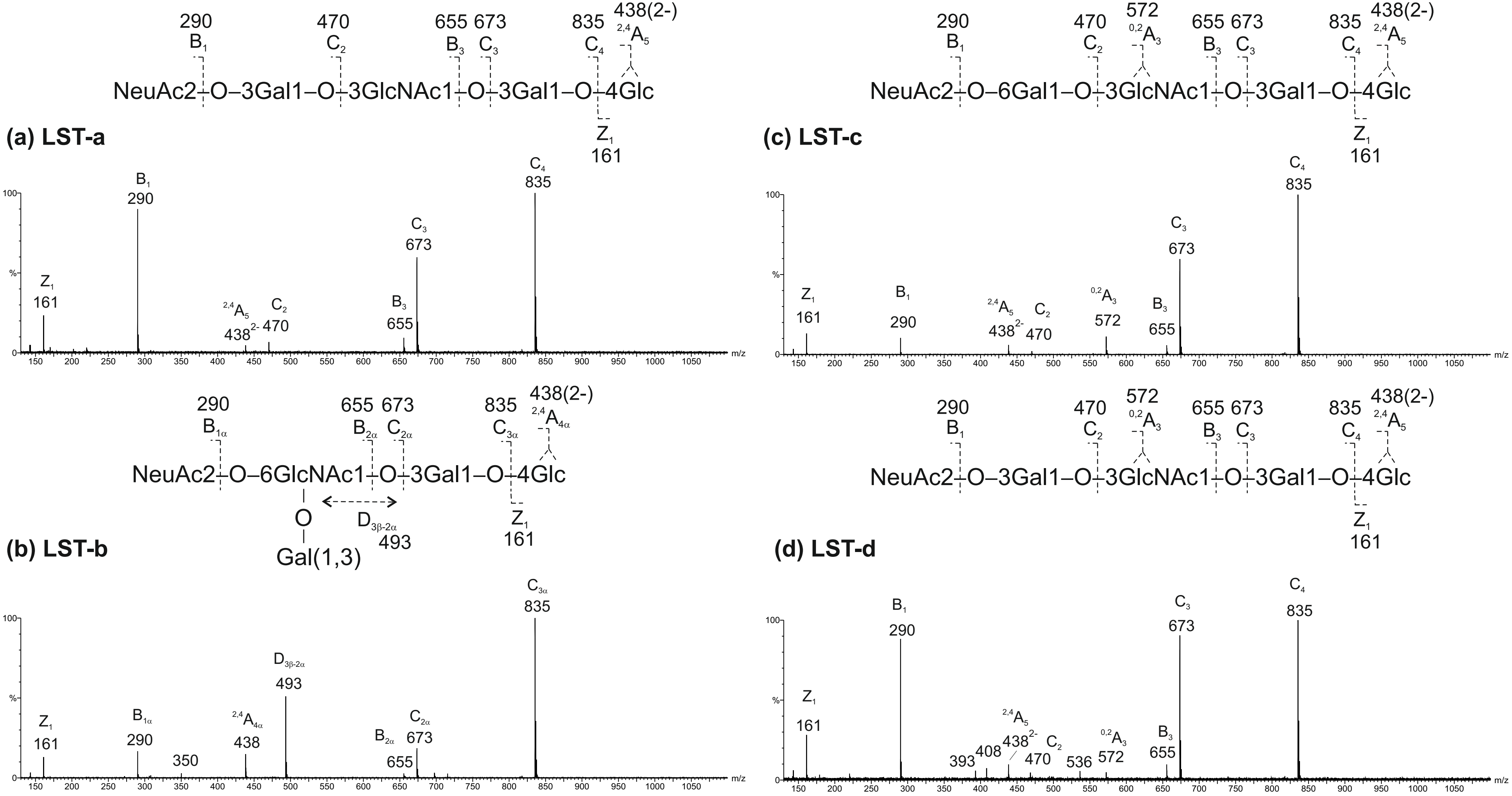

Supplementary Figure 3. ESI-CID-MS/MS of intact LSTa (a), LSTb (b), LSTc (c) and LSTd (d) using [M-2H]$^{2-}$ as the precursor ions.
